# Advances in Stem Cell Applications for Nerve Injury: A Meta-Analysis of Recent Experimental Models and Outcomes

**DOI:** 10.3390/jfb17100492

**Published:** 2026-09-29

**Authors:** Carolyn Clark, Megumi Ishii, Kyle Chen, Mengxue Xia, Ahmed Suliman, Sameer B. Shah

**Affiliations:** 1 School of Medicine, University of California San Diego, La Jolla, CA 92093, USA; 2 Division of Plastic and Reconstructive Surgery, University of California San Diego, 9500 Gilman Drive, La Jolla, CA 92093, USA; 3 Division of Biostatistics, Herbert Wertheim School of Public Health and Human Longevity Science, University of California San Diego, La Jolla, CA 92093, USA; 4 Departments of Orthopaedic Surgery and Bioengineering, University of California San Diego, 9500 Gilman Drive, MC 0863, La Jolla, CA 92093, USA; 5 Research Division, VA San Diego Healthcare System, San Diego, CA 92161, USA

**Keywords:** peripheral nerve repair, stem cells, nerve conduits, autologous nerve graft, functional recovery, meta-analysis

## Abstract

*Objective*. This meta-analysis aimed to assess the effectiveness of stem cell–seeded conduits compared with autografts and empty conduits in preclinical models of peripheral nerve repair. *Methods*. A systematic review and meta-analysis was conducted of in vivo preclinical studies published between 2013 and 2024. Endpoints included muscle mass ratio, sciatic function index (SFI), nerve conduction velocity (NCV), compound muscle action potential (CMAP) amplitude, and nerve conduction latency. Studies were further classified by stem cell type, graft material, species, and defect length. *Results*. Across 30 studies, both stem cell–seeded conduits and autografts outperformed controls across functional and electrophysiological measures. Early improvements (0–3 months) in muscle mass ratio were observed with both stem cell grafts (*p* = 0.014) and autografts (*p* = 0.027). SFI improved significantly in both groups at early time points, with stem cell grafts maintaining significance beyond 3 months (*p* = 0.04). CMAP amplitude showed early gains for both stem cell grafts (*p* < 0.001) and autografts (*p* < 0.001), with autografts performing slightly better initially (*p* = 0.033). NCV improved in both groups early, with stem cell grafts maintaining significance beyond 3 months (*p* = 0.007). Autografts showed early improvements in latency (*p* = 0.048), whereas stem cell grafts trended toward benefit without reaching significance. *Conclusions.* These findings suggest that stem cell–seeded grafts may provide functional benefits similar to autografts in peripheral nerve repair compared with empty conduits. Clinically, this method could be valuable when autografts are unavailable or when harvesting is contraindicated.

## 1. Introduction

Peripheral nerve injuries (PNIs) occur in approximately 3% of trauma patients and can significantly impair activities of daily living and overall quality of life [1]. The most common mechanism of PNI is stretching, followed by laceration, both of which can lead to long-term functional deficits if not promptly recognized and treated [2,3]. Unlike the central nervous system, the peripheral nervous system possesses the ability to regenerate; however, recovery may be hindered by factors such as gap size, slow axonal regrowth (1–3 mm per day), and prolonged denervation, with degeneration of the distal stump contributing to impaired function and end-organ atrophy [4,5]. The preferred method for optimizing recovery is tension-free peripheral nerve reconstruction; however, if a large gap (>1 cm) prevents tension-free end-to-end repair, implantation of an autologous nerve graft is the current gold standard [6,7,8].

Although autografts remain the preferred approach for peripheral nerve reconstruction, they have several limitations, including the need for additional surgical procedures with risks of infection and prolonged anesthesia, limited donor material, and challenges in finding size-matched nerves [7,8]. Additionally, donor nerves are usually limited to sensory nerves, while the injured nerves may contain both sensory and motor fibers. Donor site morbidity is another major concern; complications include neuroma formation, hypesthesia, and scar formation [7,8]. For patients who undergo autologous nerve harvesting, sensory deficits may occur in up to 92.9% of cases, chronic pain in 19.7%, and sensory symptoms in 41.1% [8].

Stem cells have increasingly been incorporated into nerve repair strategies due to their capacity to support axonal and myelin recovery. Within a regenerative environment, stem cells possess the ability to differentiate into various cell types and secrete neurotrophic factors that facilitate nerve repair [9]. Despite promising preclinical findings, stem cell–seeded grafts remain largely experimental [9].

Previous reviews of the preclinical literature have demonstrated that grafts incorporating stem cells as luminal additives exhibit regenerative potential comparable to or exceeding that of autografts while also enhancing nerve conduit vascularization [9]. However, despite increasing interest in stem cell–based approaches over the last decade, systematic reviews in the field remain limited. The last comprehensive review on the use of stem cells in peripheral nerve repair was conducted by Hundepool et al. in 2014 [10]. Given the likelihood of emerging advancements and the rapid pace of clinical translation, an updated synthesis of the literature is warranted.

This meta-analysis aims to summarize recent experimental models and trends in stem cell–seeded nerve grafts for peripheral nerve repair, focusing on studies published since 2013. Our findings suggest that stem cell–seeded conduits provide early functional benefits and achieve long-term regeneration outcomes comparable to autografts based on both functional and structural measures across animal models.

## 2. Methods

### 2.1. Identification of Studies

This meta-analysis was conducted according to the Preferred Reporting Items for Systematic Review and Meta-Analysis guidelines (PRISMA) and was not registered in a database [11]. While we evaluated some outcomes common to the previous literature, we also evaluated several new outcomes; therefore, this analysis was not considered an update of prior meta-analyses. A literature search of PubMed and Embase following PRISMA guidelines was performed. Search terms included various combinations of “stem cells,” “peripheral nerve,” “mice,” “rats,” “preclinical,” “gap,” “defect,” “conduit,” and “graft”. These terms were rearranged using Boolean operators to yield relevant papers. For example, one such search was “stem cells” AND “peripheral nerve” AND (mice OR rats) AND (gap OR defect). Another was “stem cells” AND “peripheral nerve” AND (mice OR rats) AND (“gap” OR “defect”) AND (“conduit” OR “graft”). Papers published between 2013 and 2024 were included, with the last search conducted on 17 October 2024. This review was not registered in a database, and no formal protocol was prepared prior to conducting the review.

### 2.2. Criteria for Included Studies

Refer to Table 1 for inclusion criteria based on the population, intervention, comparison, outcome, and study design (PICOS) framework. Two reviewers independently screened PubMed and Embase articles, with disagreements resolved by consensus. A Boolean search yielded 508 records; 216 duplicates were removed. Of the 292 remaining records, 46 were removed for “other reasons” as follows: studies were excluded if they were meta-analyses (*n* = 2), reviews (*n* = 8), case reports (*n* = 1), methods reports (*n* = 1), abstracts only (*n* = 30), or non-English articles (*n* = 4), resulting in 246 papers. Title screening resulted in 32 excluded studies in which stem cells were differentiated prior to implantation (*n* = 19) or irrelevant topics despite similar search terms (*n* = 13). Of the remaining 214 reports, 14 were not retrieved due to inaccessibility. After these steps, 200 articles remained.

Full-text review resulted in the exclusion of studies for the following reasons: use of stem cell–derived products or no stem cells (*n* = 55); lack of relevant endpoints or reporting only percent recovery (*n* = 49); absence of an autograft control (*n* = 36); no nerve repair (*n* = 13); no control group (*n* = 7); missing standard deviation or error (*n* = 6); missing sample size (*n* = 3); non–in vivo studies (*n* = 3); or unspecified stem cell type (*n* = 1). After this screening, 30 studies were included in the final meta-analysis. A risk-of-bias (RoB) assessment was performed on these studies using SYRCLE’s RoB tool [12]. While the included studies were scored as unbiased based on domains of baseline characteristics (criterion 2), random outcome assessment (criterion 6), and reporting bias (criterion 9), 23 of the studies were scored as biased in one or more domains of selection (sequence generation and allocation concealment), performance (random housing and blinding), detection (blinding), attrition (completeness of outcome data), and other (methodological rigor) (criteria 1, 3, 4, 5, 7, 8, and 10), primarily as a result of exclusion of any details speaking to these sources of bias in the methodology. This RoB assessment was done to understand potential limitations of conclusions associated with bias but did not ultimately impact inclusion criteria.

### 2.3. Detailed Rationale for Inclusion and Exclusion of Literature

Differentiated stem cells were excluded because lineage commitment imparts functional traits specific to target tissues [13,14]. Therefore, the question of whether differentiated stem cells, rather than undifferentiated stem cells, are comparable to autografts is a fundamentally different question and inconsistent with the aim of this meta-analysis. Most pre-differentiated cells in the literature were Schwann-like, which could bias outcomes and overestimate the regenerative potential of undifferentiated stem cells. Studies using stem cell–derived products such as exosomes, extracellular vesicles, or matrix components were similarly excluded, as these represent distinct biological entities. Additionally, studies employing alternative conduit additives, such as fibro-adipogenic progenitor cells, without direct stem cell implantation, were not considered, as they do not meet traditional stemness criteria due to lineage restriction and limited self-renewal capabilities [15].

### 2.4. Rationale for Evaluated Endpoints

Endpoints were selected based on frequency of use in assessing the efficacy of peripheral nerve repair and their inclusion in the previous meta-analysis by Hundepool et al. [10]. Studies that did not report at least one of these endpoints were excluded. Endpoints evaluated were: electrophysiological measures (nerve conduction latency, amplitude, and velocity), functional outcomes (sciatic function index [SFI]), and muscle mass ratio. Studies that reported outcomes solely as a percentage of recovered function, rather than as raw values, were excluded regardless of whether they included a relevant endpoint. Muscle mass ratio is typically reported as a percentage and therefore was excluded from this requirement.

To be included, studies had to include a control group, defined as repair without stem cells or autograft. Examples of controls included empty veins and synthetic conduits such as silicone or polylactic acid (PLA). No-repair controls were excluded, and when both a no-repair control and an empty-graft repair group existed, the empty-graft group was selected, as it offered a more relevant comparison for interventions involving graft implantation.

**Figure 1:**
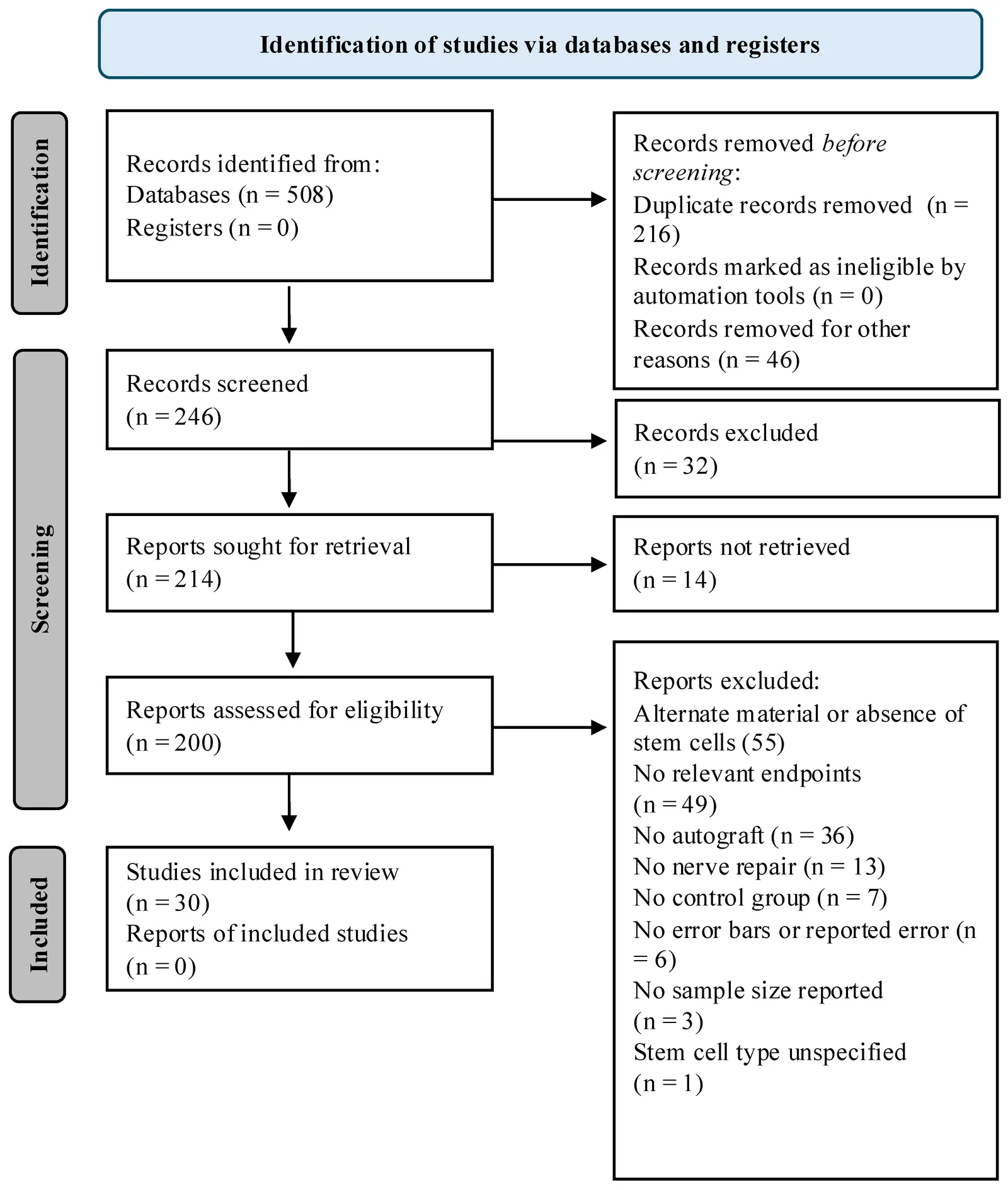
Flow diagram for systematic review. In total, 508 articles were found in the search. After screening as described above, a total of 30 articles were included in this study. The PRISMA Checklist is found in the Supplementary Materials section.

Several studies were excluded because they used bone defect models and did not incorporate nerve repair. Studies using stem cells only *in silico* or *in vitro* were also excluded. For example, Zaminy et al. (2021) assessed the effect of detergents on adipose-derived stem cell survival in decellularized grafts but did not perform in vivo nerve repair [16]. All exclusion criteria are summarized in Figure 1. Eligible papers were assigned to reviewers for data extraction; no automated screening tools were used.

### 2.5. Data Collection and Extraction

Data extraction was performed by a designated reviewer for each article, with reviewers assigned a specific number of articles to ensure equitable distribution of the work. To verify the accuracy of the extracted data, all entries were independently verified by a separate reviewer. When numerical values were not provided in the articles, they were extrapolated using Plot Digitizer, a validated online data extraction tool [17].

Categorical variables were collected for stem cell lineage, graft material, experimental species, animal sex, nerve gap length, and observation duration. Treatment efficacy was evaluated according to the endpoints outlined above. For comparative analysis, studies were divided into two groups according to follow-up interval: those with observation periods of 0–3 months and those exceeding 3 months. If an article reported multiple values due to measurements at various time points within each period, the value from the last time point was used for analysis.

### 2.6. Data and Statistical Analysis

Statistical analysis was conducted using R version 4.4.1. Data sets that reported standard deviation (σ) were converted to standard error with the formula: *standard error* = *σ*/(√*n*). Data sets lacking SEM, SD, or error bars were excluded from the final analysis (*n* = 6). Pie charts were generated to display all categorical variables. Outcome data were synthesized using random-effects meta-analysis models to account for biological and methodological heterogeneity across studies. Heterogeneity was quantified using the I^2^ statistic. Forest plots were created in R version 4.4.1 from the collected outcome data. For tests of difference, *p* < 0.05 was considered statistically significant. Plots displayed estimates along with 95% confidence intervals.

## 3. Results

### 3.1. Distribution of Stem Cell Type

**Figure 2:**
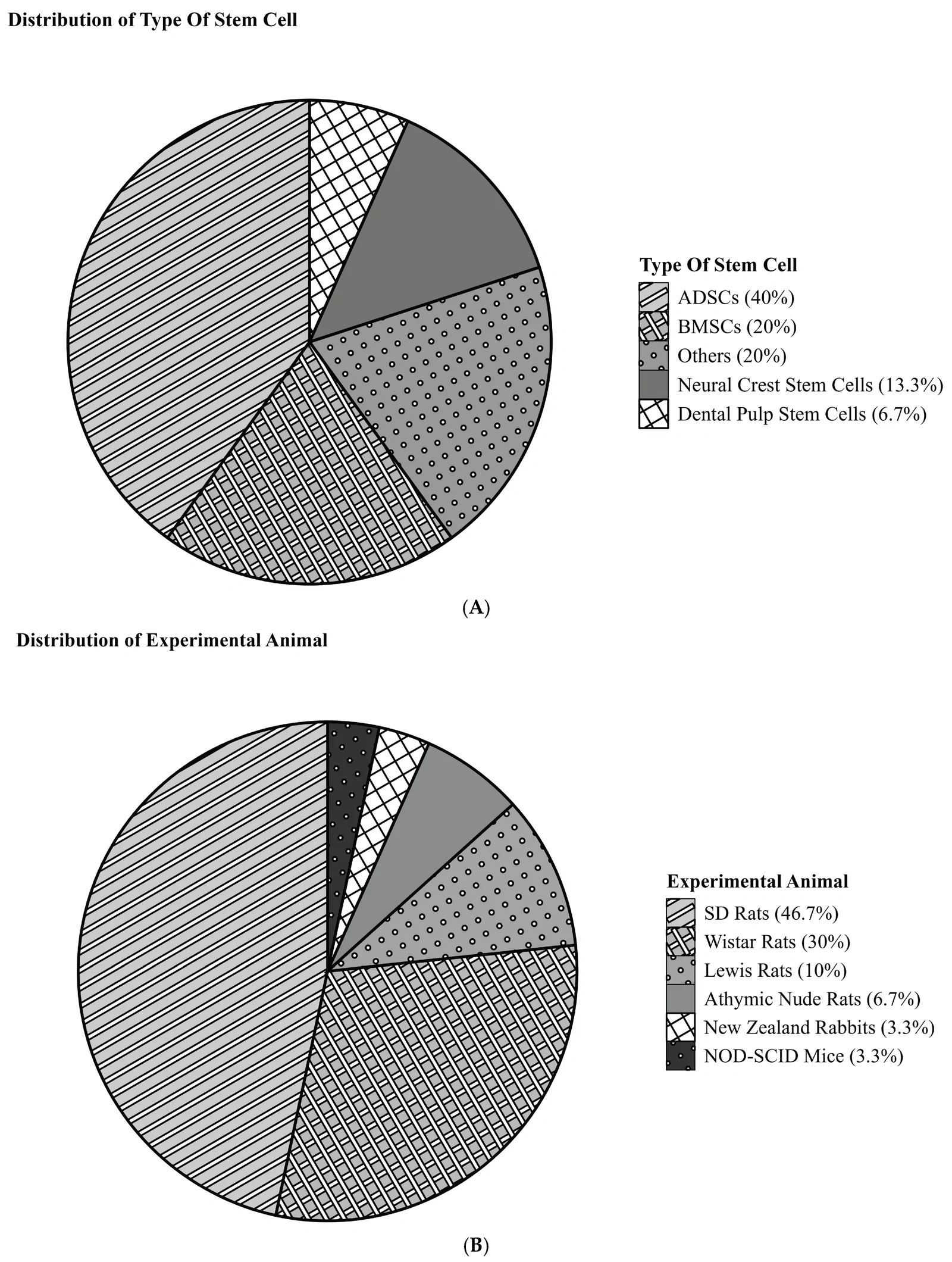

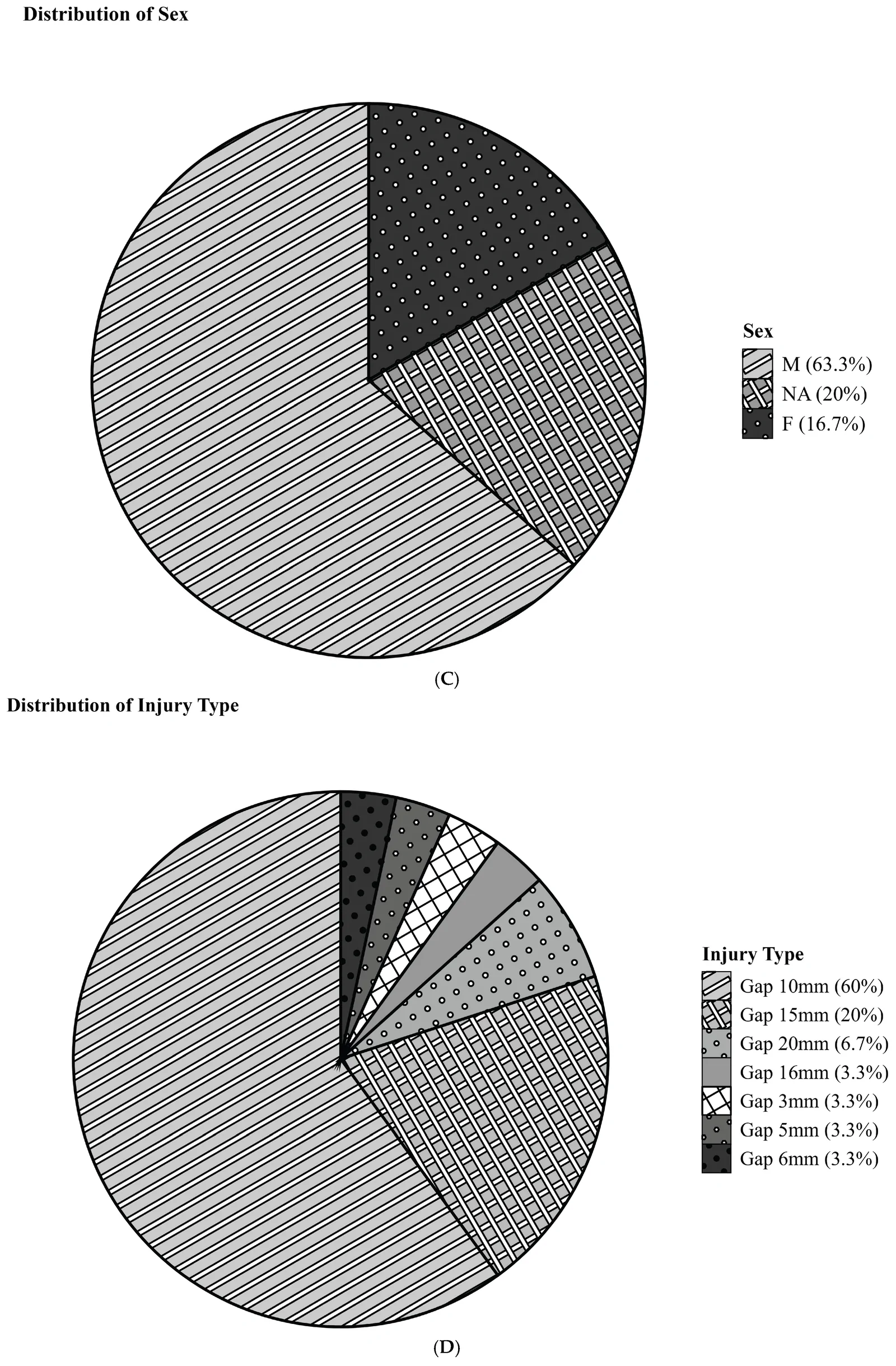

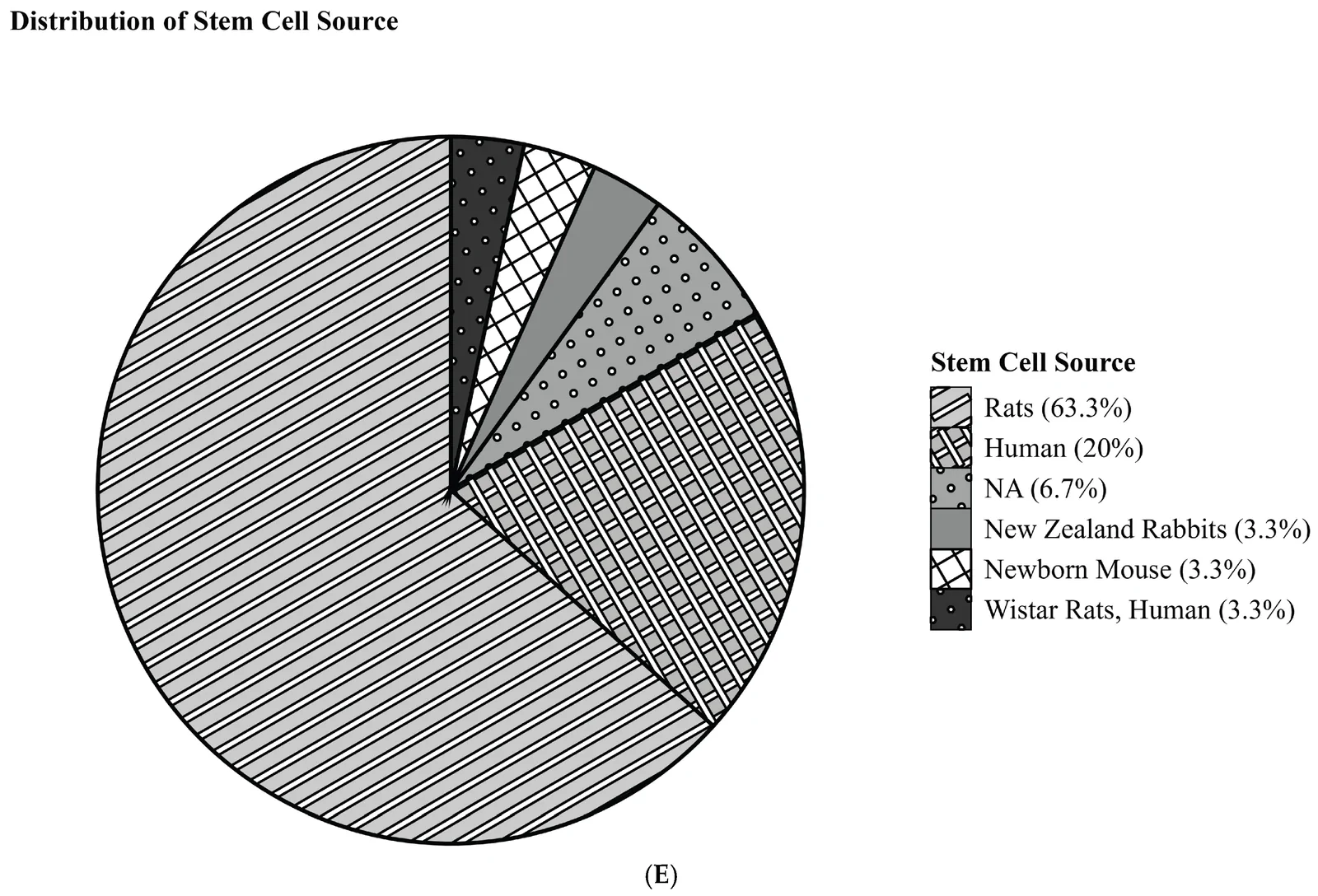
Pie charts showing distribution of various study characteristics. (**A**) Stem cell type. (**B**) Experimental animal. (**C**) Experimental animal sex. (**D**) Nerve gap length. (**E**) Stem cell source.

This meta-analysis found that, over the past decade, adipose-derived stem cells (ADSCs) were the most frequently used stem cell type for peripheral nerve repair. ADSCs accounted for 40% of the included studies, followed by bone marrow–derived stem cells (BMSCs) at 20%, neural crest stem cells at 13.3%, and dental pulp stem cells at 6.7%. The remaining 20% comprised single studies using exfoliated deciduous teeth stem cells, menstrual blood stem cells, olfactory-derived stem cells, umbilical cord blood–derived stem cells, endothelial stem cells, and amniotic fluid–derived stem cells (Figure 2A).

### 3.2. Experimental Animal Models

Sprague-Dawley rats comprised 46.7% of the studies, Wistar rats 30%, Lewis rats 10%, athymic rats 6.7%, New Zealand rabbits 3.3%, and NOD-SCID mice 3.3% (Figure 2B). Among these models, 63.3% used male animals, and 16.7% used female animals. Sex was not specified in the remaining 20% of studies (Figure 2C). Gap-type injury (nerve transection) was the only injury type included in our analysis, although this was not an intentional limitation of the study (Figure 2D).

### 3.3. Stem Cell Source

Stem cells were derived from various sources, with rats being the most common (63.3%), followed by humans (20%), New Zealand rabbits (3.3%), and mice (3.3%). One study utilized both rat and human stem cells. Two studies did not specify stem cell source (Figure 2E).

### 3.4. Muscle Mass Ratio

**Figure 3:**
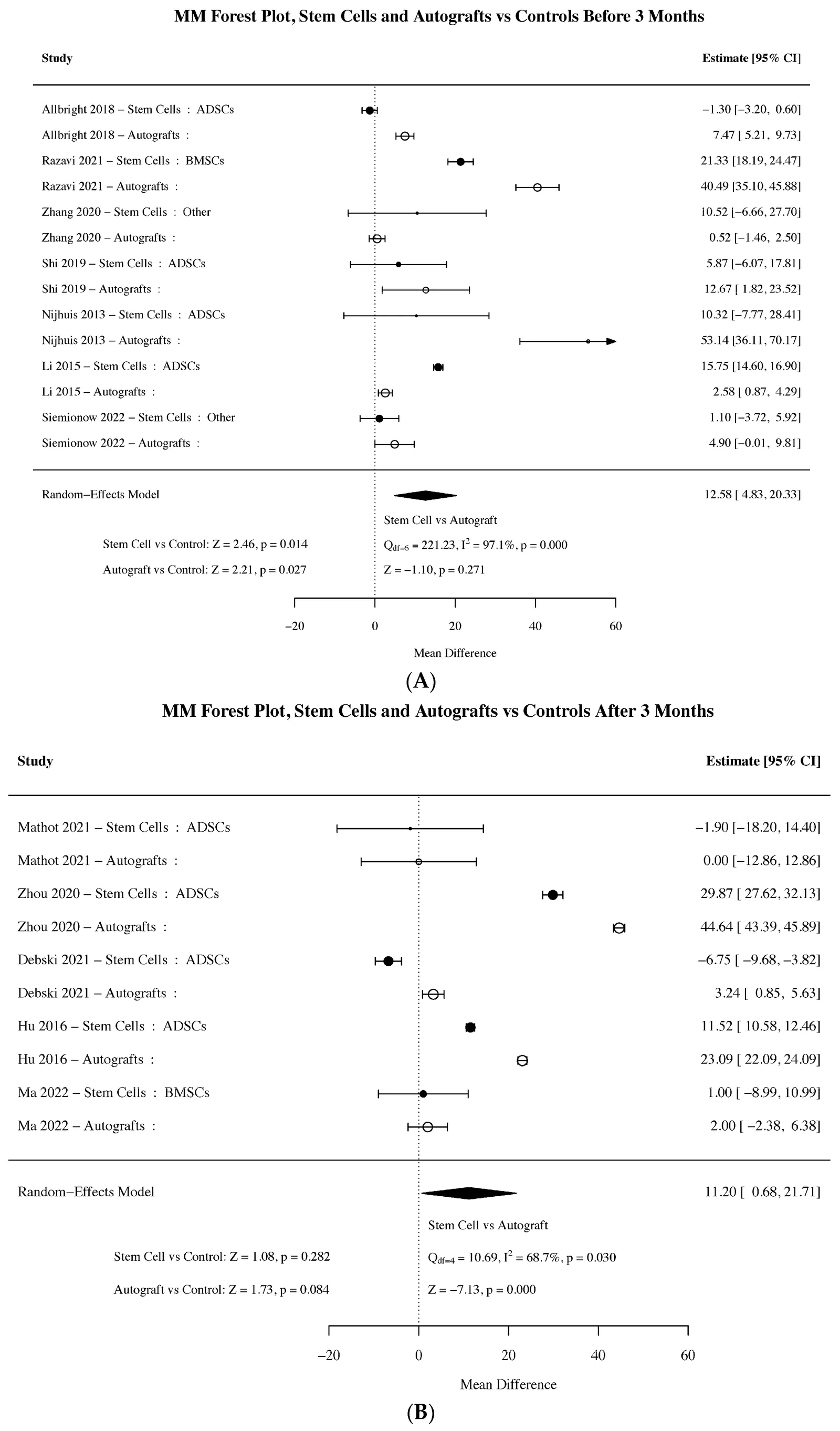
(**A**) Forest plot summarizing the effect sizes of included studies for muscle mass ratio between 0 and 3 months post-operation [18,19,20,21,22,23,24]. (**B**) Forest plot summarizing the effect sizes of included studies for muscle mass ratio >3 months post-operation [25,26,27,28,29]. Closed circles denote stem cell conditions, and open circles denote autografts. Larger symbols represent studies with more data/weight and smaller symbols represent studies with less data/weight. Plots display individual study estimates with confidence intervals, pooled effect sizes, and heterogeneity (I^2^).

Muscle mass ratio (MM ratio), an indicator of muscle preservation after nerve injury, was measured in 12 studies and showed significant early improvements with both stem cell–seeded grafts and autografts. Between 0 and 3 months, stem cell–treated conditions exhibited a significant increase in MM ratio compared with controls (Z = 2.46, *p* = 0.014), with autografts showing a similar effect (Z = 2.21, *p* = 0.027). However, at later time points (>3 months), neither intervention retained significance. Stem cells showed a diminished effect (Z = 1.08, *p* = 0.282), while autografts trended toward improvement but did not reach significance (Z = 1.73, *p* = 0.084). These findings suggest that while both interventions promote early muscle preservation, their long-term impact on muscle mass retention may be limited (Figure 3).

### 3.5. Sciatic Function Index

**Figure 4:**
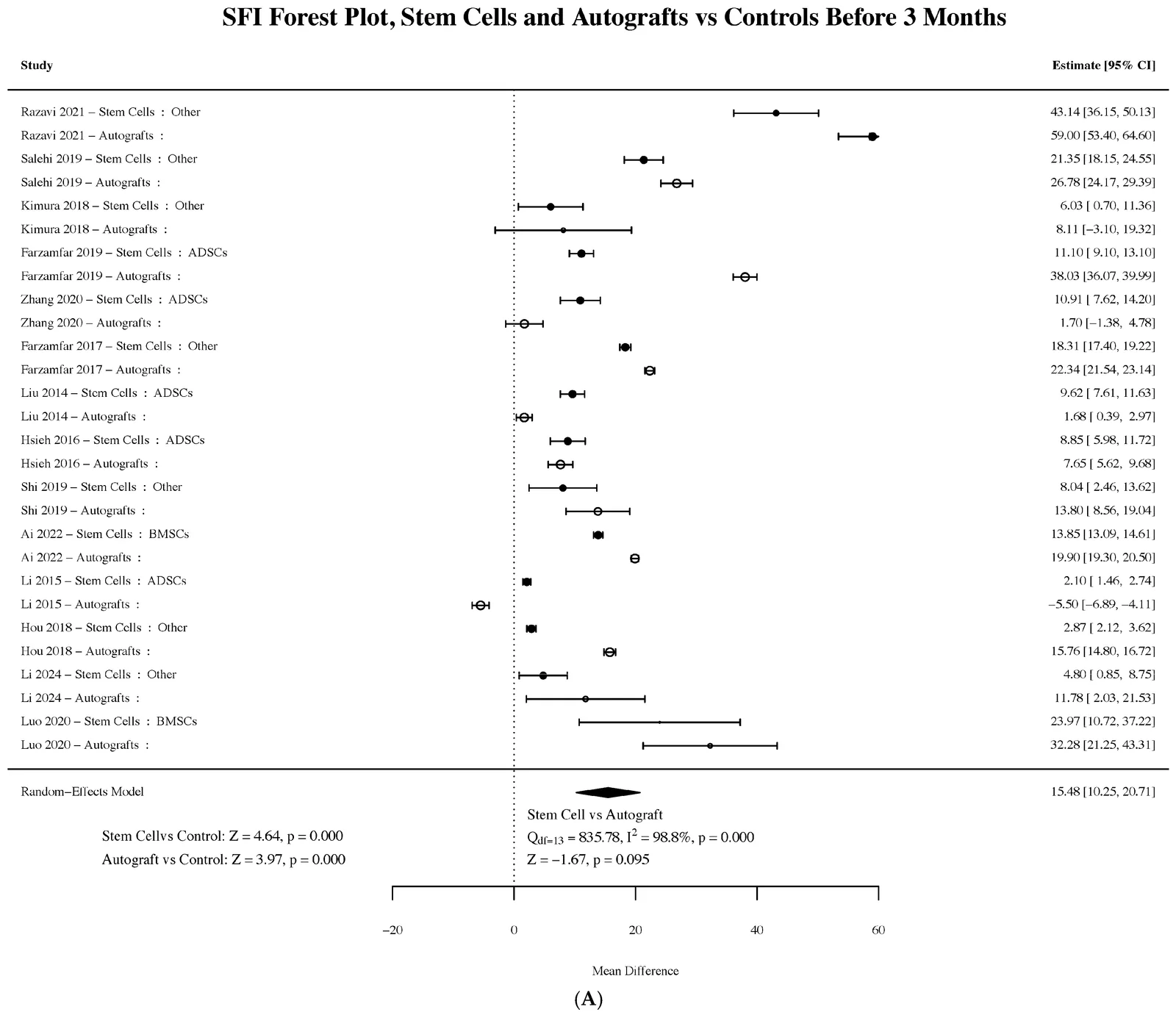

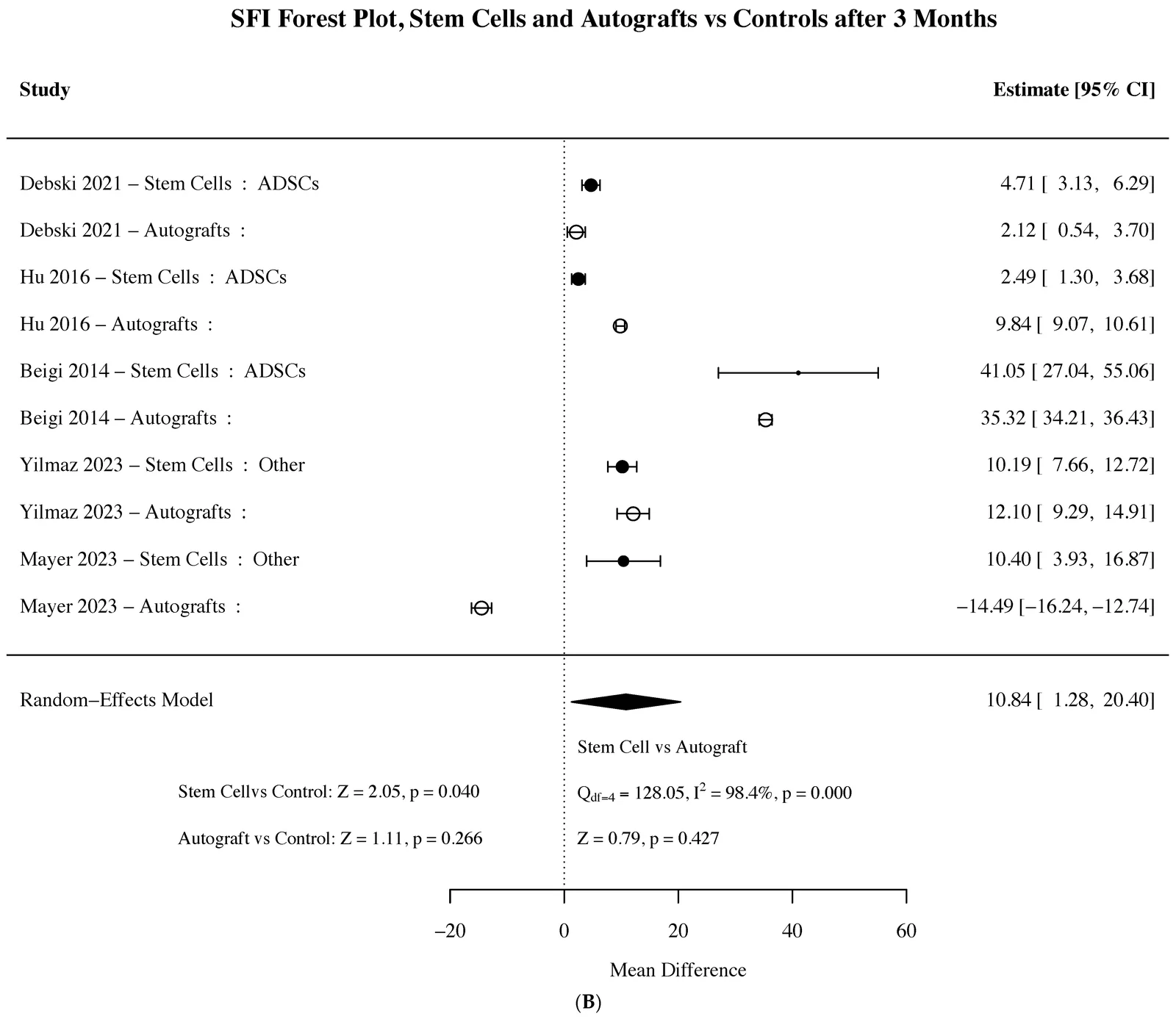
(**A**) Forest plot summarizing the effect sizes of included studies for SFI between 0 and 3 months post-operation [19,20,21,23,30,31,32,33,34,35,36,37,38,39]. (**B**) Forest plot summarizing the effect sizes of included studies for SFI >3 months post-operation [27,28,40,41,42]. Closed circles denote stem cell conditions, and open circles denote autografts. Larger symbols represent studies with more data/weight and smaller symbols represent studies with less data/weight. Plots display individual study estimates with confidence intervals, pooled effect sizes, and heterogeneity (I^2^).

The sciatic function index (SFI) was reported in 19 of the 30 studies included in the analysis. Stem cell–seeded grafts and autografts demonstrated comparable improvements in performance relative to control repairs at both early (0–3 months) and later (>3 months) time points. During the first three months, both interventions demonstrated similar improvements in SFI compared to controls, with stem cell–seeded grafts exhibiting a Z-score of 4.64 (*p* < 0.001) and autografts a Z-score of 3.97 (*p* < 0.001). At later time points (>3 months), stem cell conditions retained significant improvements versus controls (Z = 2.05, *p* = 0.04), while autograft conditions did not (Z = 1.11, *p* < 0.26) (Figure 4).

### 3.6. Amplitude

**Figure 5:**
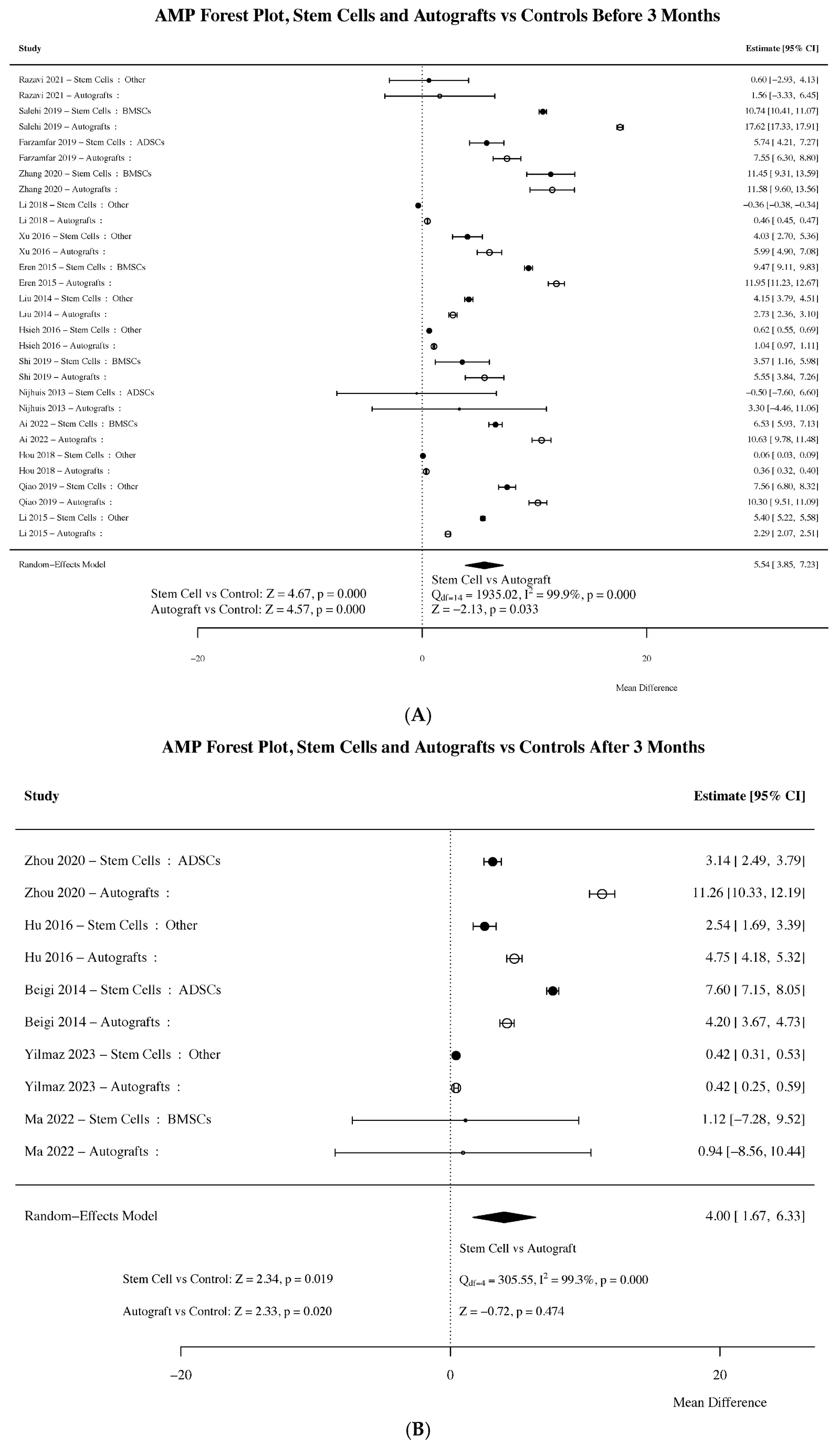
(**A**) Forest plot summarizing the effect sizes of included studies for action potential amplitude between 0 and 3 months post-operation [19,20,21,22,23,30,31,34,35,36,37,43,44,45,46]. (**B**) Forest plot summarizing the effect sizes of included studies for action potential amplitude >3 months post-operation [26,28,29,41]. Closed circles denote stem cell conditions, and open circles denote autografts. Larger symbols represent studies with more data/weight and smaller symbols represent studies with less data/weight. Plots display individual study estimates with confidence intervals, pooled effect sizes, and heterogeneity (I^2^).

Compound muscle action potential (CMAP) amplitude was reported in 20 of the 30 studies. Both autografts and stem cell–seeded grafts demonstrated similar and significant improvements compared to controls. Between 0 and 3 months, increases in CMAP amplitude outcomes were superior to controls for both stem cell–seeded grafts (Z = 4.67, *p* < 0.001) and autografts (Z = 4.57, *p* < 0.001). Autografts showed a slightly improved outcome between 0 and 3 months compared to stem cell–seeded grafts (*p* = 0.033). After 3 months, both stem cell–seeded grafts (Z = 2.34, *p* < 0.019) and autografts (Z = 2.33, *p* < 0.020) maintained significant improvement relative to controls but not each other (Figure 5).

### 3.7. Nerve Conduction Velocity

**Figure 6:**
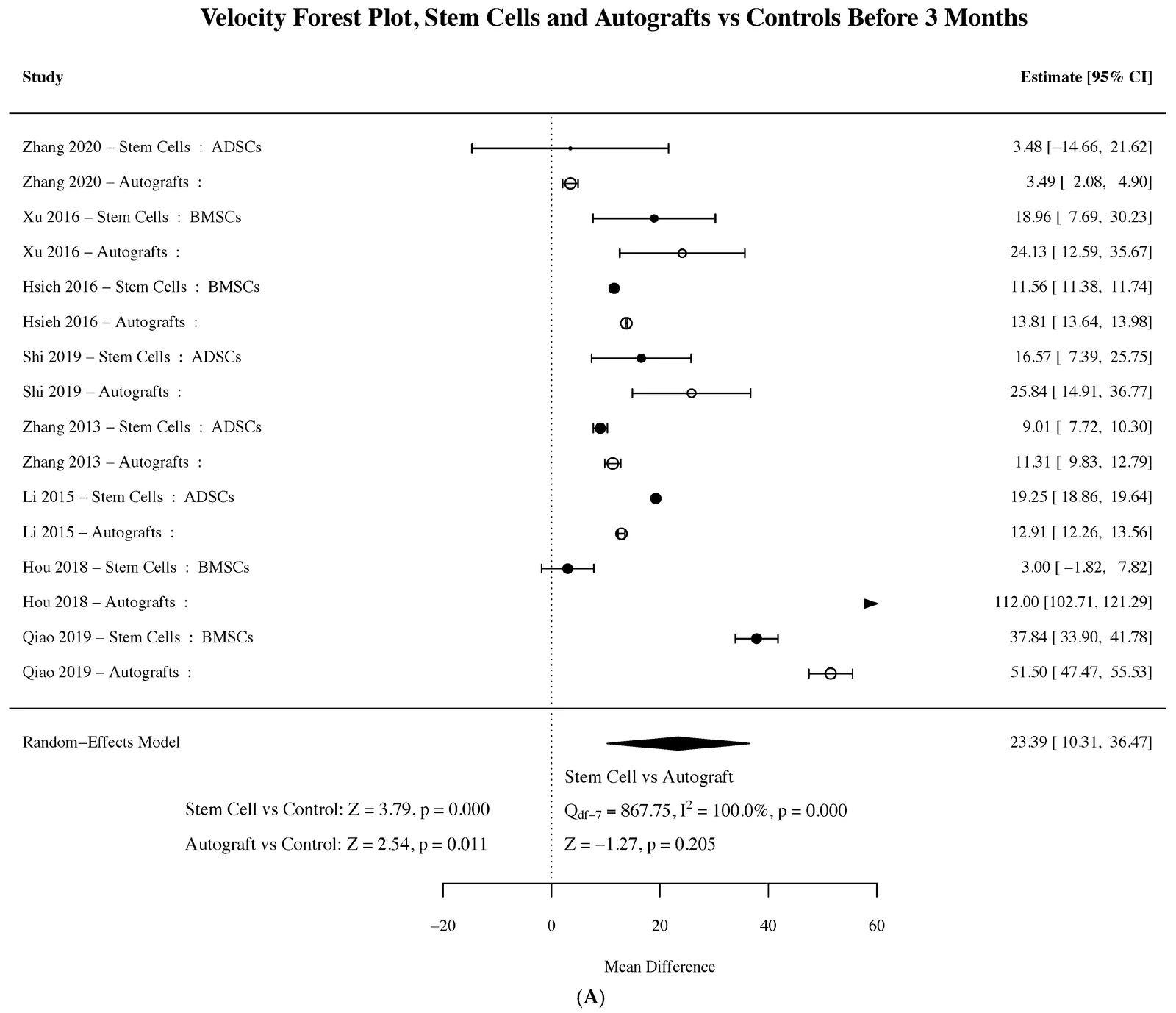

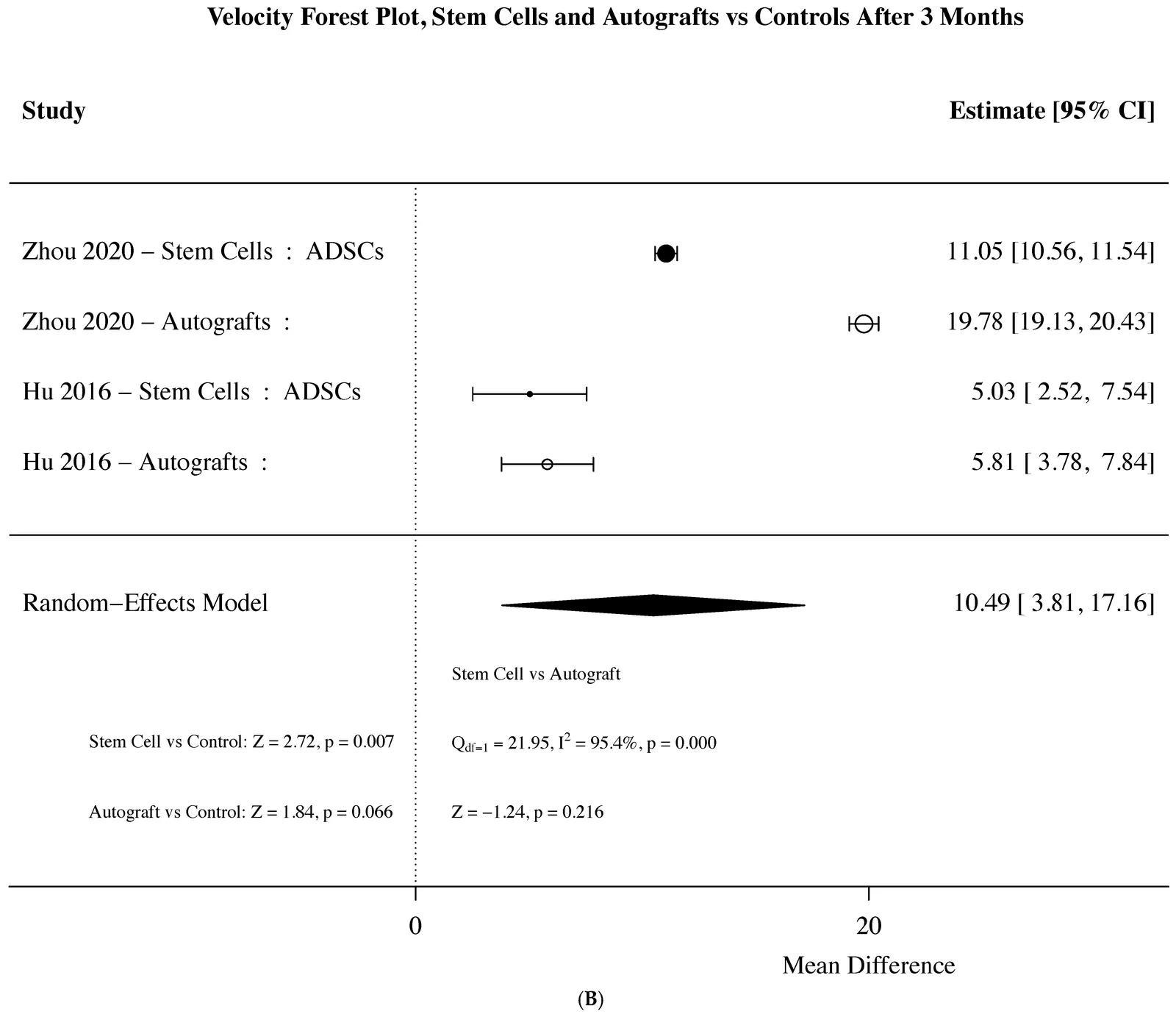
(**A**) Forest plot summarizing the effect sizes of included studies for action potential velocity between 0 and 3 months post-operation [20,21,23,35,37,44,46,47]. (**B**) Forest plot summarizing the effect sizes of included studies for action potential velocity >3 months post-operation [26,28]. Closed circles denote stem cell conditions, and open circles denote autografts. Larger symbols represent studies with more data/weight and smaller symbols represent studies with less data/weight. Plots display individual study estimates with confidence intervals, pooled effect sizes, and heterogeneity (I^2^).

Nerve conduction velocity (NCV) was measured in a subset of studies (10 out of 30) and showed significant improvement with both stem cell–seeded grafts and autografts compared to controls, particularly in the early post-repair period. Between 0 and 3 months, NCV was significantly higher in both the stem cell group (Z = 3.79, *p* < 0.001) and the autograft group (Z = 2.53, *p* = 0.011) compared to controls. After 3 months, NCV remained elevated in the stem cell group (Z = 2.27, *p* = 0.007), while autografts showed a smaller, nonsignificant trend toward improvement (Z = 1.84, *p* = 0.066) (Figure 6).

### 3.8. Latency

**Figure 7:**
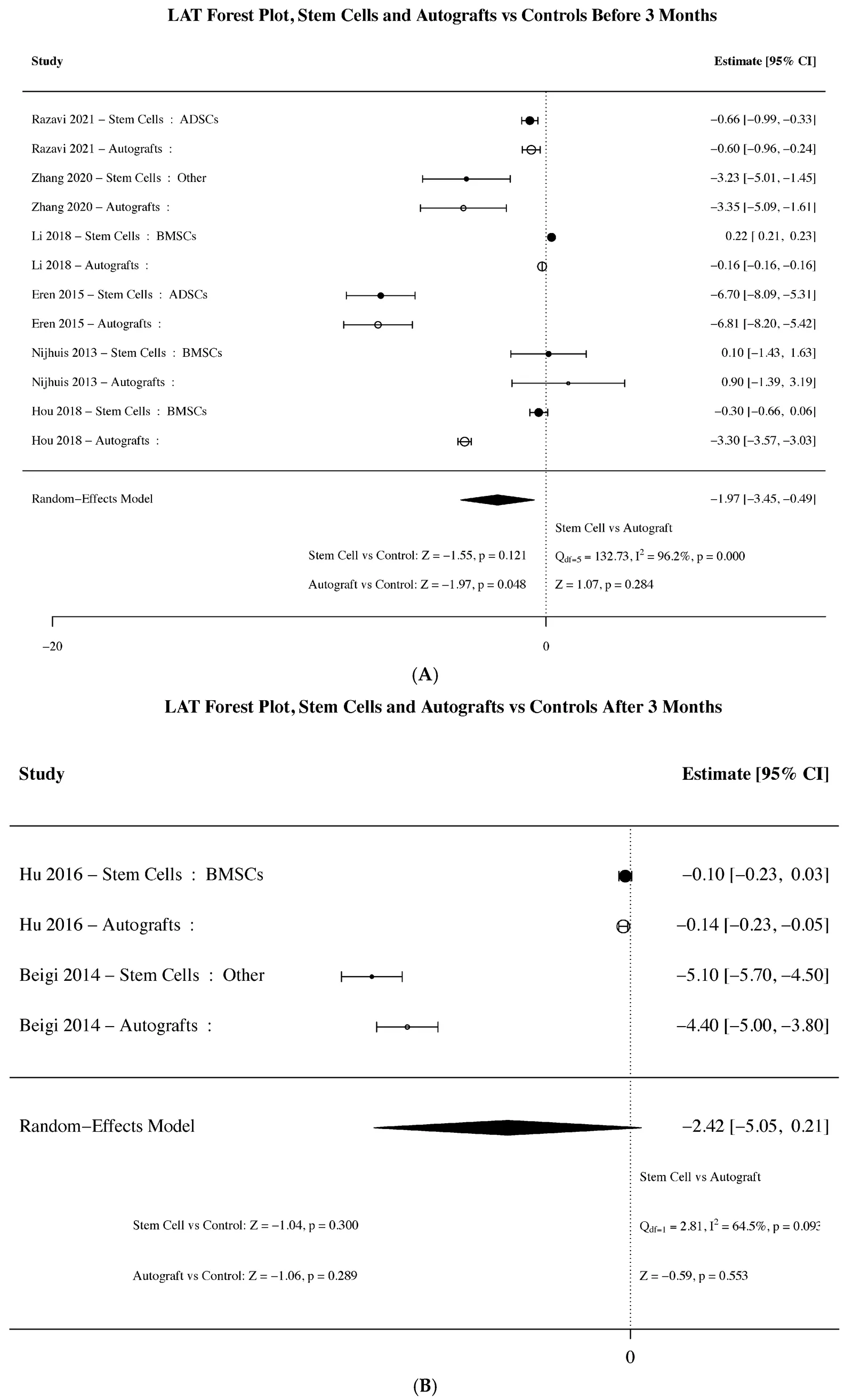
(**A**) Forest plot summarizing the effect sizes of included studies for action potential latency between 0 and 3 months post-operation [19,20,22,37,43,45]. (**B**) Forest plot summarizing the effect sizes of included studies for action potential latency >3 months post-operation [28,40]. Closed circles denote stem cell conditions, and open circles denote autografts. Plots display individual study estimates with confidence intervals, pooled effect sizes, and heterogeneity (I^2^).

Latency, a measure of nerve conduction efficiency, was assessed in 8 out of 30 studies and showed variable results over time. Between 0 and 3 months, autografts demonstrated a modest yet significant reduction in latency compared to controls (Z = −1.97, *p* = 0.048), while stem cell–seeded grafts exhibited a similar trend that did not reach significance (Z = −1.55, *p* = 0.121). At later time points (>3 months), latency improvements in the stem cell group were further diminished (Z = −1.06, *p* = 0.289) (Figure 7).

## 4. Discussion

This meta-analysis demonstrates that stem cell–seeded nerve grafts achieve functional outcomes comparable to autologous grafts, with both approaches showing early benefits in peripheral nerve repair. These findings highlight stem cell–based strategies as a potential alternative when autograft use is limited or contraindicated while also avoiding the morbidity associated with graft harvesting.

### 4.1. Trends in Preclinical Models Used to Evaluate Stem Cells for Nerve Regeneration

Hundepool et al. (2014) reported that stem cells used for peripheral nerve repair were mostly represented by bone marrow stem cells (BMSCs) [10]. However, the most commonly used luminal additive has shifted from BMSCs to ADSCs. This may be due to ADSCs’ relative accessibility, ease of harvest, and differentiation potential. Compared to BMSCs, ADSCs can be harvested with lower clinical risk, as the procedure can be performed under local anesthesia with minimal discomfort [48,49]. The diversity of stem cell sources, even within this small sample size, reflects ongoing efforts to identify the optimal cell type for nerve repair. However, the limited representation of certain populations such as umbilical cord blood, menstrual blood, and amniotic fluid–derived stem cells demonstrates the need for further investigation into their efficacy.

Consistent with studies conducted before 2013, there has been minimal evolution in the selection of animal models, with rats being used in 93.4% of studies included in this analysis [10]. The predominance of Sprague-Dawley and Wistar rats aligns with their established use in nerve injury models, providing consistency across preclinical models. However, male animals are overrepresented (63.3%), potentially limiting the generalizability of findings, as sex-based biological differences could influence nerve regeneration outcomes. While rats are ideal models for evaluating feasibility and proof of concept, they have unusually strong regenerative capacity and face additional size constraints, limiting the achievable nerve gap sizes [50].

Kaplan et al. (2015) note that murine models, with their small defects, may skew treatment outcomes and fail to represent the larger injuries seen in humans (5–30 cm) [51]. This limitation is reflected in the experimental models included in this analysis, as the largest gap studied was 2 cm. Our meta-analysis reinforces that there remains an unmet need for the development of a preclinical model that more closely mirrors the scale and complexity of human nerve injuries to better evaluate the translational potential of emerging therapies. Rabbit models, which were the only large animal used to evaluate stem cells in the evaluated studies, may represent such a model [52].

Additionally, Kaplan et al. note that current research may prioritize technologies that perform well in rat models while prematurely discontinuing those that do not. This approach could overlook treatments that might be effective in nerve injury models that more closely reflect clinical conditions [51]. For example, all studies included in this analysis focused exclusively on nerve gap injuries. While this may reflect an emphasis on the clinical challenges associated with this injury type, it also restricts the applicability of results to other forms of nerve damage, such as crush, compression, traction (stretch), or partial transection. Traction injuries, the most common type of traumatic nerve injury, were not present in any of the studies included in this analysis [2,3].

### 4.2. Efficacy of Stem Cell–Based Nerve Regeneration Strategies

Functional recovery relies on restoration of both sensorimotor control and axonal regeneration. While structural metrics such as axon count, myelination, and vascular patency inform regeneration, functional assessments better capture overall recovery, with preclinical studies emphasizing motor recovery due to its slower clinical progress compared with sensory recovery [53,54]. For instance, Petersen et al. (2017) linked improvements in motor-evoked potentials and CMAPs with better upper-extremity functionality in spinal cord injury patients [53]. Similarly, Korte et al. (2011) showed that serial electrophysiologic recordings in rats could reliably track axonal regeneration and motor recovery after peripheral nerve injury [54]. Together, these findings support the use of such metrics as indicators of functional recovery.

Electrophysiological outcomes, which encompass the nerve conduction velocity (NCV), action potential amplitude, and nerve conduction latency, offer high-resolution examinations of neuromuscular function. Both stem cell and autograft interventions significantly improve CMAP amplitude early on and maintain statistical significance beyond 3 months, supporting their role in neuromuscular recovery. Both interventions demonstrate improvements in nerve conduction velocity in the early phase, suggesting enhanced early myelination or axonal regeneration. Consistent with findings by Hundepool et al. [10], our data show that stem cells significantly enhance CMAP amplitude and NCV compared to non-cell controls.

Notably, our analysis did not reveal a correlation between NCV and latency, which is unexpected given their theoretical inverse relationship. One explanation is the limited overlap between studies reporting both metrics; only four out of the 14 studies contributing to these categories measured both NCV and latency, potentially reducing the ability to detect meaningful associations. This underscores the importance of more consistent reporting of outcomes in future studies to allow for more meaningful comparisons.

The second functional outcome assessed was the sciatic function index (SFI), a simple, noninvasive measure reflecting recovery of complete neuromuscular circuits. Consistent with electrophysiological findings, early improvements (<3 months) in SFI suggest that stem cell–seeded grafts accelerate functional recovery compared to controls. However, only stem cell conditions remained significant beyond 3 months, indicating that stem cell therapy may provide persistent functional benefits. Similar to Hundepool et al. [10], our findings demonstrate that stem cell–seeded conduits improve SFI outcomes in early recovery and sustain benefits beyond 3 months.

However, SFI is a relatively low-resolution metric derived from footprint length and toe spread and may not capture nuanced aspects of gait recovery such as compensatory walking adaptations or contractures [55]. For example, one study found no correlation between tibialis anterior muscle force and SFI after autograft repair, although ankle angle during toe-off did correlate with force generation [55]. Therefore, while our results highlight the potential of stem cell therapies to accelerate and prolong functional gains, the limited resolution of SFI warrants the integration of more precise outcome measures to confirm functional improvements.

A subset of studies evaluated muscle mass ratio as a measure of motor reinnervation after peripheral nerve injury. Consistent with Hundepool et al. [10], our findings suggest that stem cell–based interventions and autografts improve muscle mass ratio in the early stages of recovery. However, we observed that this benefit declines over time, ultimately losing significance. This temporal pattern suggests that while these interventions may offer transient protection against muscle atrophy, they do not ensure sustained motor reinnervation. It is important to note, however, that the relatively small number of studies reporting muscle mass ratio (*n* = 12) and heterogeneity in methodology may limit the statistical power to detect effects, and the observed decline could reflect this limitation. Like SFI, muscle mass is used because of its simplicity; however, more detailed assessments of reinnervation, while more technically demanding, are advisable [56].

Cumulatively, findings from electrophysiological, SFI, and muscle mass outcomes are consistent with the notion that late phases of functional recovery are less dependent on successful regeneration across the injury site, which primarily occur within the early phase of recovery. Rather, they may reflect improvements in the stabilization and maturity of regenerated neurons as well as improved communication between regenerated neurons and their motor targets. Such improvements are consistent with morphological changes (e.g., higher axon counts, re-myelination, and reduced fibrosis) noted in regenerating nerves. A basis for these changes was not explored, but may reflect the activity of exosomes or extracellular vesicles generated by the stem cells, as recently reviewed [57,58,59].

### 4.3. Limitations

This meta-analysis had several limitations. One important limitation was the small sample size (30 studies), which precluded subgroup or sensitivity analysis, introduced higher variability into the data, and decreased statistical power. This limitation was compounded by the non-standardized experimental design among these studies, including differences in stem cell source, culture conditions, conduit material, animal species, nerve gap length, and follow-up duration, leading to our inclusion only of the terminal time point in each study to allow clustering and subsequent comparison. This heterogeneity in design as well as several ambiguities in the presentation of methodology contributed to substantial bias in over 75% of the studies (noted through the RoB assessment), despite similar trends in all compared studies. Moreover, because the maximum nerve gap length studied was 2 cm, the results cannot be directly extrapolated to larger defects typically seen in clinical practice. The predominance of young, male animals in current studies may also limit generalizability, underscoring the need to address sex- and age-related differences in regenerative capacity in future research. From an analytical standpoint, we deployed mean difference across Forest plots, to enable comparison of pooled outcomes at broadly defined early and late time points, despite heterogeneous experimental conditions and differences in post-repair time points. This approach mirrored that used by Hundepool et al. [10] An alternative strategy would have been to use standardized mean difference-based methods (e.g., Hedges’ *g*), which account for scale variance for a common outcome across studies; such an approach was considered but not implemented given the pooling of disparate outcomes. Finally, given the above limitations, the present analysis could not reliably assess superiority (or lack thereof) between stem cell–based approaches and autografts. Future studies should include larger, standardized studies with consistent experimental design and reporting of SFI, electrophysiological metrics, and muscle mass ratio at defined time points, supplemented by morphological analyses or biological tissue characterization.

## 5. Conclusions

Our findings suggest that stem cell–seeded grafts may provide early functional and electrophysiological benefits similar to autografts in peripheral nerve repair. Clinically, this approach could be useful when autograft supply is limited or otherwise contraindicated. This strategy also reduces morbidity associated with autograft harvesting. However, due to the small sample size and variability among studies and multi-factorial sources of bias, these results should be interpreted with caution. Systematic research is needed to confirm these findings and optimize stem cell–based therapies for clinical application.

## Figures and Tables

**Table 1:** Inclusion criteria based on PICOS framework.

PICOS	Inclusion Criteria
Population	Peripheral nerve injury in animal model
Intervention	Peripheral nerve injury repaired with stem cell–seeded non-autologous nerve graft
Comparison	Autograft alone and conduit alone
Outcome	Muscle mass ratio, sciatic function index, nerve conduction velocity, compound muscle action potential amplitude, nerve conduction latency
Study Design	In vivo animal study, English language
Year of Publication	2013–2024

## Data Availability

The original contributions presented in this study are included in the article. Further inquiries can be directed to the corresponding authors.

## Supplementary Materials

The following supporting information can be downloaded at: https://www.mdpi.com/article/10.3390/jfb17100492/s1. Reference [60] is cited in the Supplementary Materials.
